# Intestinal removal of free fatty acids from hosts by *Lactobacilli* for the treatment of obesity

**DOI:** 10.1002/2211-5463.12024

**Published:** 2016-01-18

**Authors:** Hea‐Jong Chung, Jae G. Yu, In‐Ah Lee, Ming‐Jie Liu, Yan‐Fei Shen, Satya P. Sharma, Mohammad A. H. M. Jamal, Jun‐Hyun Yoo, Hyeon‐Jin Kim, Seong‐Tshool Hong

**Affiliations:** ^1^ Department of Biomedical Sciences and Institute for Medical ScienceChonbuk National University Medical SchoolJeonjuChonbukKorea; ^2^ JINIS BDRD instituteJINIS Biopharmaceuticals Co.WanjuChonbukKorea; ^3^ Department of Family MedicineSamsung Medical CenterSungkyunkwan University School of MedicineSeoulKorea

**Keywords:** absorption, free fatty acid, intestinal, *Lactobacillus*, obesity

## Abstract

Recent findings on the association of gut microbiota with various diseases, including obesity, prompted us to investigate the possibility of using a certain type of gut bacteria as a safe therapeutic for obesity. *Lactobacillus* mutants with enhanced capacity in absorption of free fatty acids (FFAs) were isolated to show reduced absorption of FFAs by the administered host, attributing to inhibition of body weight gain and body fat accumulation as well as amelioration of blood profiles. Consequently, high throughput screening of natural FFAs‐absorbing intestinal microbes led to the isolation of *Lactobacillus* *reuteri* JBD30 l. The administration of *Lactobacillus* JBD30l lowered the concentration of FFAs in the gut fluid content of small intestine, thus reducing intestinal absorption of FFAs whereas promoting fecal excretion of FFAs. Animal data also confirmed that the efficacy of *Lactobacillus* JBD30l on body weight similar to that of orlistat, an FDA‐approved pharmaceutical for long‐term use to treat obesity. In a subsequent random, double‐blind, placebo‐controlled clinical trial (KCT0000452 at Clinical Research Information Service of Korea), there was a statistically significant difference in the percentage change in body weight between the *Lactobacillus* JBD301 and the placebo group (*P* = 0.026) as well as in the BMI (*P *= 0.036) from the 0‐week assessment to the 12‐week assessment. Our results show that FFA‐absorbing *Lactobacillus* JBD301 effectively reduces dietary fat absorption, providing an ideal treatment for obesity with inherent safety.

**Abbreviations**
BMI: body mass index
CFU: colony‐forming unit
CNS: central nervous system
CT: computerized tomography
DEXA: dual‐emission X‐ray absorptiometry
DIO: diet‐induced obesity
FFAs: free fatty acids
GI: gastrointestinal
HDL: high‐density lipoprotein cholesterol
HFD: high‐fat diet
LDL: low‐density lipoprotein cholesterol
MRI: magnetic resonance imaging
MRS: Man–Rogosa–Sharpe
NTG: *N*‐methyl‐*N*‐nitro‐*N*‐nitrosoguanidine
SCFA: short‐chain fatty acids
SD: Sprague–Dawley
TC: total cholesterol
TG: triacylglycerol
USFA: unsaturated fatty acids

Obesity is a disease of energy balance, characterized by a chronic disequilibrium between energy intake and expenditure 1. It is associated with an increased risk of various chronic diseases, including hypertension, dyslipidemia, type 2 diabetes, cardiovascular disease, obstructive sleep apnea, osteoarthritis and cancer 2. Because obesity is rarely curable and its prevalence has increased over several decades, intensive research has been conducted to develop anti‐obesity drugs, resulting in many candidates with very interesting anti‐obesity effects at preclinical levels 3, 4, 5. The path to drug development for obesity, however, has been littered with failures in clinical development and withdrawals from the market due to severe side effects 6, 7, 8. Orlistat, the major FDA‐approved pharmaceutical for long‐term use to treat obesity, is a gastric and pancreatic lipase inhibitor that prevents fat hydrolysis, thus reducing dietary fat absorption by ~ 30% 9, 10, 11. Despite substantial anti‐obesity effects, the inhibition of lipase activity by orlistat generates undigested fat in the gastrointestinal (GI) tract, which causes side effects that are not only uncomfortable but also socially unacceptable 12.

Unlike orlistat, several appetite suppression drugs were successfully approved and marketed for obesity treatment, but these drugs have been withdrawn due to severe psychiatric and/or cardiovascular side effects, all common adverse effects of central nervous system (CNS)‐acting drugs 13. The FDA recently approved two new anti‐obesity drugs that work on the CNS, lorcaserin and phentermine/topiramate, driven by high demand for anti‐obesity drugs 14, 15, 16; however, the future of these new drugs remains uncertain, considering the history of anti‐obesity drugs that work on the CNS. Because of the safety issues with anti‐obesity drugs that work on the CNS 17, anti‐obesity drugs with different modes of action are under investigation 18, 19. Drugs targeting pathways in the metabolic tissues, the peptidergic signaling systems of hunger and satiety, and the homeostatic mechanisms related to leptin have shown potential in preclinical studies, but none of the drugs has been safe and effective in clinical development thus far. Therefore, a new type of anti‐obesity treatment must be actively sought because the current pharmaceutical drugs are not ideal for the treatment of obesity.

A new paradigm is obviously necessary to develop anti‐obesity drugs engendering sustained weight loss with minimal side effects. Recent evidence showed that the gut microbiota play an intricate role in the regulation of body weight 20, 21, 22. Transplantation experiments using microbiotas from either obese or lean mice and from either obese or lean humans into germ‐free mice proved that the compositional change in microbiota in the GI tract resulted in differences in the efficiency of caloric extraction from food, eventually contributing to different body weights 21, 22. These results suggest that small changes in caloric extraction from the GI tract by transplanting a specific intestinal bacterium can lead to a meaningful reduction in body weight. In fact, attempts have been made to identify specific intestinal bacterium to control obesity. Interestingly, a few probiotic strains have been shown to ameliorate obesity and metabolic disorders, without clear understanding of underlying mechanisms 23, 24.

Dietary fat is the major contributor in our caloric extraction from food. Because fat is degraded into FFAs before absorption into the body, the removal of FFAs in the GI tract by the transplantation of a FFAs‐absorbing bacterium might be an ideal choice for treating obesity by decreasing fat uptake by the host body. Transplanted microbes with enhanced capacity in FFA absorption would compete for FFAs with enterocytes in the intestinal epithelium, resulting reduced FFA absorption and thus lowered caloric intake into host. In fact, recent study have shown that microbiota contribute obesity by stimulating intestinal FFA absorption in the zebrafish model, suggesting inhibition of intestinal FFA absorption and lipid droplet formation to regulate host obesity 25. Considering that increased FFA along with hyperglycemia are the key hallmarks of obesity, metabolic syndrome and diabetes, FFA provides an excellent metabolic target to reduce dietary energy harvest and thus prevent and counteract obesity. In addition, reduction in caloric extraction with FFAs‐absorbing bacteria may be a better choice than inhibiting fat hydrolysis by orlistat, which results in an unavoidable problem with undigested fat.

Based on the fact that orlistat, which inhibits digestion of dietary fat to FFAs, is currently the best anti‐obesity drug, as well as the fact that small changes in caloric extraction affected by intestinal microbiota could lead to large body weight differences, we investigated whether the inhibition of FFAs absorption into the human body by using an intestinal bacterium would lead to the development of anti‐obesity drugs producing sustained weight loss without side effects. As expected, the administration of FFAs‐absorbing lactobacilli that remove intestinal FFAs before absorption to host showed significant anti‐obesity effects, with efficacy as high as orlistat in animal experiments and a clinical trial. Our results not only provide a novel Lactobacillus approach as a safe way to prevent or treat obesity but also suggest the feasibility of developing treatments by modulating the metabolic activities of the intestinal microbes.

## Materials and methods

### Materials

Reagents and kits were purchased from Sigma, except for the following: [1‐^14^C]‐palmitic acid (PerkinElmer Life Sciences, Santa Clara, CA, USA), liquid scintillation cocktail (LSC, PerkinElmer Life Sciences), [carboxyl‐^14^C]‐triolein (PerkinElmer Life Sciences), Man–Rogosa–Sharpe (MRS, Becton Dickinson, NJ, USA), orlistat (Xenical, Roche, Basel, Switzerland), EnzyChrom^™^ Free Fatty Acid Assay Kit (BioAssay Systems, Hayward, CA, USA). The sterilizable 384‐well plate and 384‐pin replicator were from Nunc. The membrane semidry system was from Bio‐Rad, and the X‐ray film was from Kodak. The gel‐pro analyzer software was from Media cybernetics. The magnetic resonance imaging (MRI) images were obtained with a Bruker Biospec 47/40 4.7‐Tesla instrument (Bruker, Billerica, MA, USA) and analyzed with image j software (NIH, Bethesda, MD, USA). The serum was analyzed with a Rat/Mouse ELISA kit (LINCO research, St. Charles, MO, USA), a Leptin ELISA kit (R&D System, Minneapolis, MN, USA), a blood glucose meter (Accu‐Chek, Roche) and Cholesterol ELISA kits (Asan Pharmaceutical, Seoul, Korea).

### Isolation of FFAs‐absorbing *Lactobacillus* mutants

*Lactobacillus acidophilus* KCTC 3179 is a human‐derived *Lactobacillus* strain from the Korea Collection for Type Cultures (KCTC, Daejeon, Korea). Anaerobic culture was carried out in an anaerobic jar (BBL Gas‐Pack anaerobic systems, Apeldoorn, Netherland). *Lactobacillus acidophilus* KCTC 3179 were chemically mutagenized by using *N*‐methyl‐*N*‐nitro‐*N*‐nitrosoguanidine (NTG) to select the *Lactobacillus* mut1 followed by 2nd‐round mutagenesis by using 4‐nitroquinoline‐1‐oxide to select *Lactobacillus* mut2 that absorbs FFAs strongly. FFAs‐absorption ability of the mutants was evaluated *in vitro* by measuring radioactivity with liquid scintillation spectrophotometry after incubation for 30 min with ^14^C‐labeled palmitic acid. For *in vivo* evaluation, Sprague–Dawley (SD) rats had been fed standard diet (60% complex carbohydrates, 22% protein, 3.5% fat, 5% fiber, 8% crude ash, 0.6% calcium and 1.2% phosphorus) supplemented with testing mutants at 10^7^ CFU·day^−1^ for 8 weeks. Intestinal colonization of mutants was confirmed by counting. Then, blood at 2 h, 4 h, 6 h, 8 h and 10 h were analyzed for radioactivity after feeding ^14^C‐labeled triolein whereas FFAs were analyzed with fecal fluid content with GC/MS.

### Animal experiments

All animal care and use were performed strictly in accordance with the ethical guidelines by the Ethics Committee of Chonbuk National University Laboratory Animal Center and the animal study protocol was approved by the institution (CBU No. 2012‐0040).

### Anti‐obesity effects of FFAs‐absorbing *Lactobacillus* mutants

The anti‐obesity effects were assessed under diet‐induced obesity (DIO) condition with SD rats fed high‐fat diet (HFD, 48% complex carbohydrates, 17.6% protein, 22.8% fat, 4% fiber, 6.4% crude ash, 0.48% calcium, and 0.96% phosphorus) supplemented with testing mutants at 10^7^ CFU·day^−1^ for 22 weeks. The body weights of the SD rats were measured weekly. At the end of the experiments, MRI analysis was performed to measure the visceral (or abdominal) fat with a Bruker Biospec 47/40 4.7‐Tesla instrument. Blood samples from the experimental rats were collected with overnight fasting at 0 and 22 weeks. The sera were analyzed for biochemical characteristics using commercially available kits. The serum total cholesterol (TC), high‐density lipoprotein cholesterol (HDL), low‐density lipoprotein cholesterol (LDL) levels as well as the triacylglycerol (TG) concentrations were detected with the ELISA kits described in the Reagents section above.

### Identification of intestinal FFAs‐absorbing *Lactobacillus* JBD301

*Lactobacillus* strains were isolated from the feces of healthy lean adult volunteers. For individual lactobacilli overnight cultures, the quantities of FFAs in the cultured media were determined using the EnzyChrom^™^ free fatty acid assay kit (Bio‐Assay Systems, Hayward, CA, USA) as described by the manufacturer. Using the concentration of FFAs in the media, the quantity of FFAs uptake from the media was calculated. A natural *Lactobacillus* strain with the strongest FFAs‐absorbing ability was identified, taxonomically classified and phylogenetically analyzed by 16S rDNA sequences (NCBI GenBank, DNA data bank of Japan, European Nucleotide Archive, www.phylogeny.fr).

### Animal studies of *Lactobacillus* JBD301

Seven‐week‐old female C57BL/6 mice were fed HFD supplemented daily with 10^7^ CFU of *Lactobacillus* JBD301 for 3 weeks. Then, gut fluid contents were collected and analyzed for total FFAs concentration at small intestine and large intestine of the mice with the EnzyChrom^™^ FFAs assay kit. To evaluate changes in FFAs absorption and excretion by host fed *Lactobacillus* JBD301, the radioactivities of the bloods as well as feces were measured after feeding 1 μCi of ^14^C‐labeled triolein.

### Clinical trial of *Lactobacillus* JBD301

A phase 2, double‐blind, placebo‐controlled human study was conducted with *Lactobacillus* JBD301 at the Samsung Medical Center (Seoul, Korea). The research protocol was approved by the Clinical Trial Center at the Samsung Medical Center and the methods were carried out in accordance with the approved guidelines (SMC No. 2011‐04‐061‐002). This trial was registered with Clinical Research Information Service of Korea as KCT0000452 (https://cris.nih.go.kr). The purpose of this study was to evaluate the safety and tolerability of *Lactobacillus* JBD301 and to evaluate the efficacy of *Lactobacillus* JBD301 compared to placebo for the reduction in body fat or weight in adults with 25 ≤ BMI < 35. Among the recruited subjects, 37 subjects were randomly assigned to either the placebo group or experimental group. Placebo group (*n* = 19) were administered single capsule of vegetable cream as control whereas experimental group (*n* = 18) were administered *Lactobacillus* JBD301 at dose of 1 × 10^9^ CFU·day^−1^ for 12 weeks. For the clinical data, the statistical analysis was performed using procedures in sas (Version 9.2) and medcalc (Version 11.6.0). Depending on the normality of the underlying data, the Mann–Whitney *U* test and the Wilcoxon signed rank test were used to perform statistical analysis.

Further details of the materials and methods used in this study can be found in the supplementary material.

## Results

### *Lactobacillus* mutants with enhanced FFAs absorption reduced FFAs absorption by host

**Figure 1:**
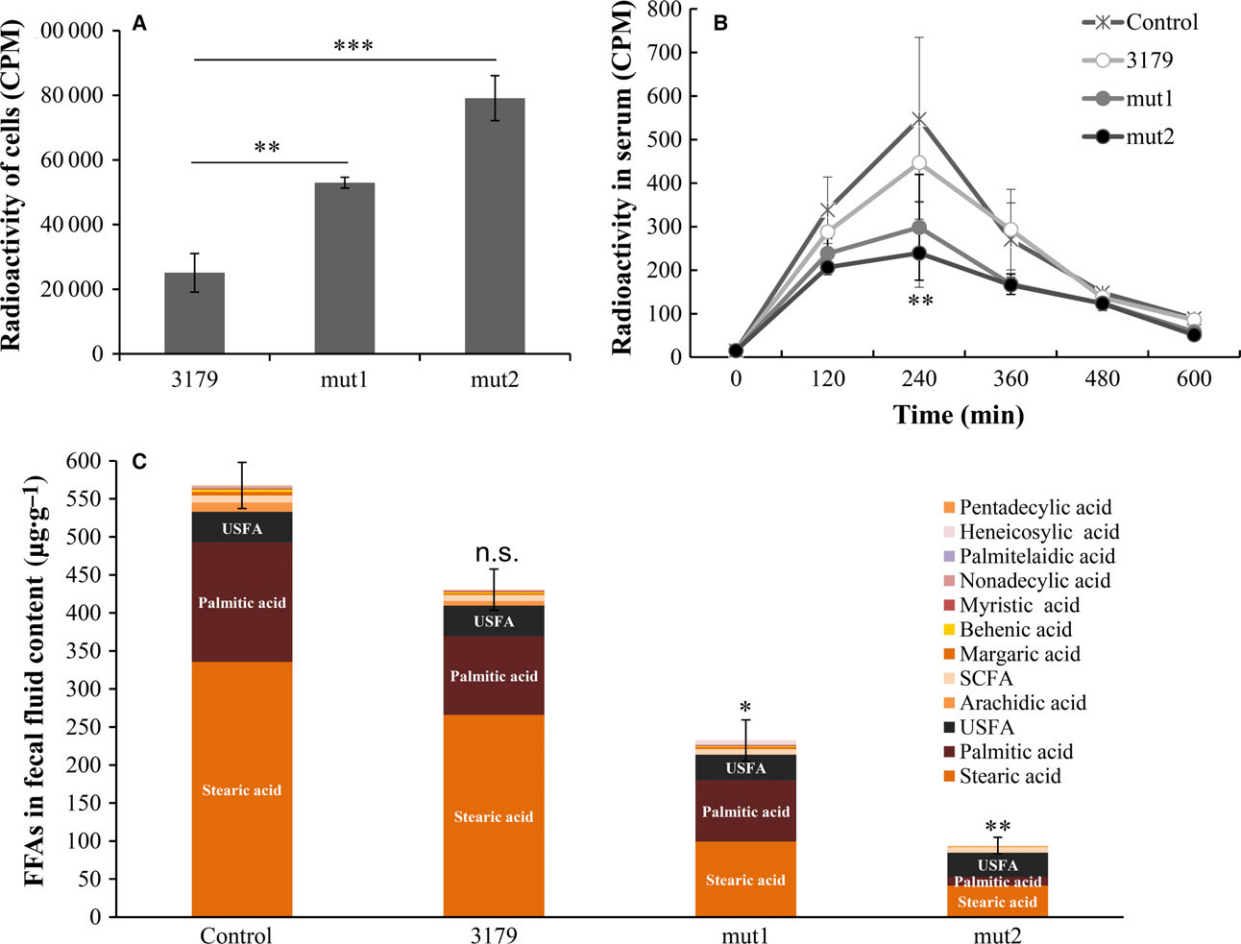
Administration of the FFAs‐absorbing *Lactobacillus* mutants significantly reduced the absorption of FFAs by the host. (A) The FFAs absorption by lactobacilli (*L. acidophilus* KCTC 3179, mut1 or mut2) was determined by measuring the radioactivity in lactobacilli after incubation with ^14^C‐labeled palmitic acid for 30 min. Values are mean ± SD (*n* = 4). (B) Three‐month‐old male SD rats were randomized and fed a standard diet only (control) or a diet‐supplemented daily with 10^7^ CFU of tested lactobacilli (*L. acidophilus* KCTC 3179, mut1 or mut2). After 8 weeks, rat feed containing radiolabeled fat, ^14^C‐triolein, was administered. Blood samples were collected at the indicated times and radioactivity was analyzed to quantify the amount of FFAs from absorbed dietary fat in the blood of hosts. Values are mean ± SD (*n* = 9). (C) FFAs in the fecal fluid contents from the hosts were analyzed by GC/MS. Major saturated fatty acids, stearic acid and palmitic acid, were indicated in the bar. Unsaturated Fatty Acid (USFA) includes oleic acid, arachidonic acid. Short‐chain Fatty Acid (SCFA) includes acetic acid, propanoic acid, butanoic acid, valeric acid, pentanoic acid, hexanoic acid. Values are mean ± SEM (*n* = 4). Statistical significance is shown as *P < 0.05, **P < 0.01 and ***P < 0.001 versus control by ANOVA.

To test the hypothesis that obesity can be controlled by an intestinal bacterium that removes obesity‐causative metabolites, i.e. FFAs, in the gut, a commercial probiotic strain, *L. acidophilus* KCTC 3179, was mutagenized by NTG to isolate mutants that strongly absorb palmitates, the most common form of FFAs. We initially isolated a mutant that absorbed 2.1 times more ^14^C‐palmitate from its surrounding environment than the parental strain (Fig. 1A). The identified mutant, mut1, was further mutagenized by 4‐nitroquinoline 1‐oxide, resulting in mut2, which absorbed FFAs 3.2 times more strongly than the parental strain (Fig. 1A). For the identified mutants, the acidification abilities during growth and colonization in the host GI tract after consumption were examined because these are the most important characteristics of edible *Lactobacillus* 26. Both mutant strains showed normal growth and acidification activities during milk fermentation (Table S1). They also successfully colonized the GI tract of rats (Table S2). These results indicate that both mutants function as normal lactobacilli, except for their stronger absorption of FFAs.

When consuming lactobacilli, the bacteria transiently colonize the small intestine 27, where most of FFAs are absorbed into the body. Therefore, the administered mutants could actively remove intestinal FFAs by functioning as a bio‐sequestrant, reducing the amount of FFAs available to be absorbed into the host body and thereby reducing dietary fat absorption. To test whether the mutants actually reduce the amount of absorbable FFAs in the GI tract of the host, SD rats were fed *Lactobacillus* for daily administration of 10^7^ CFU for 8 weeks. After colonization, rat feed containing radiolabeled dietary fat, ^14^C‐triolein, was orally administered to the rats so that the amount of FFAs absorbed from dietary fat could be measured by measuring the radioactivity of the FFAs in their blood (Fig. 1B). Compared to the high‐fat diet (HFD) control, the rats colonized with the both mutant strains showed a significant decrease in the amount of absorbed FFAs, whereas those colonized with parental strain 3179 showed no significant change. At 240 min after administration of radiolabeled dietary fat, the radioactivity of absorbed FFAs in the blood of the rat colonized with mut1 and mut2 were down to 298.4 cpm and 206.6 cpm, whereas control and parental strain 3179 group were 546.9 and 446.6 cpm respectively. As shown in Fig. 1B, the rats colonized with mut1 and mut2, but not parental strain, absorbed significantly less FFAs than the uncolonized controls.

If those mutants absorb FFAs strongly in the GI tract and thus remove FFAs available for the GI tract of the host, the FFAs quantities in the feces should decrease in the rats colonized with the mutants. We performed GC/MS analysis on the fecal fluid contents from *Lactobacillus*‐fed rat, as described previously 28. As expected, the total FFAs quantities in the fecal fluid content were significantly lowered in the rats colonized with the mutants compared to the uncolonized control whereas parental strain show some reduction (Fig. 1C). Most dramatic changes were with saturated fatty acids, particularly, stearic acid and palmitic acid. Unsaturated fatty acids (USFA), such as oleic acid and arachidonic acid were also reduced as well as short‐chain fatty acids (SCFA), such as acetic acid, propanoic acid, butanoic acid, valeic acid, pentanoic acid and hexanoic acid. Particularly, pentadecanoic acid, palmitelaidic acid, nonadecanoic acid, heptanoic acid, heneicosanoic acid, sebacic acid, dodecanoic acid were reduced to undetectable ranges in the mut2 group. This overall decrease in FFAs including short‐chain FA in the liquid portion of the fecal matter indicates that the mutants actively absorbed and removed intestinal FFAs in the colonized GI tract as shown in fecal fluid, thereby reducing the amount of FFAs available to be absorbed by the host.

### FFAs‐absorbing *Lactobacillus* mutants ameliorated obesity in rats

**Figure 2:**
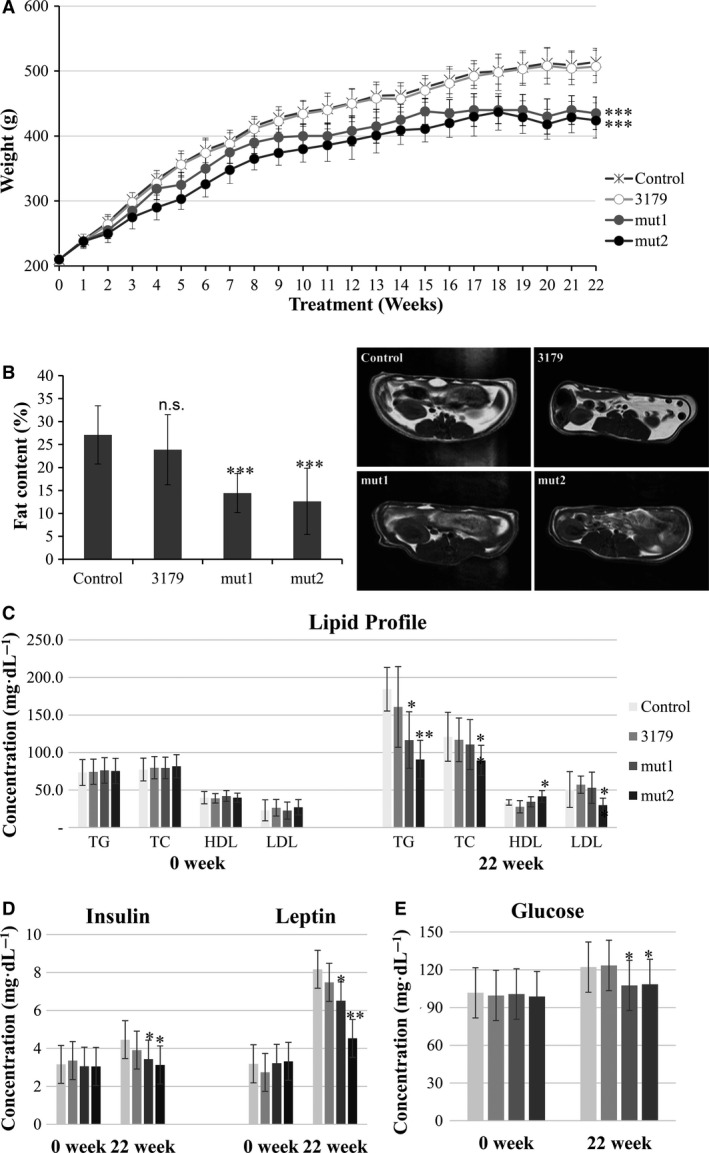
FFAs‐absorbing *Lactobacillus* mutants effectively inhibited weight gain and body fat accumulation and also ameliorated blood profiles under diet‐induced obesity condition. Three‐month‐old male SD rats were randomized and fed a HFD only (control) or a HFD diet supplemented daily for 22 weeks with 10^7^ CFU of tested lactobacilli (*L. acidophilus* KCTC 3179, mut1, or mut2). (A) Body weights were monitored weekly. (B) The change in visceral fat areas of the rats. The representative MRI images of visceral fat accumulation were shown. (C) The change in plasma lipid profiles. TG, triacylglycerol; TC, total cholesterol; HDL, high‐density lipoprotein cholesterol; LDL, low‐density lipoprotein cholesterol. (D) The change in plasma insulin and leptin concentrations. (E) The change in blood glucose concentrations. Values are mean ± SD (*n* = 14). Statistical significance is shown as *P < 0.05, **P < 0.01 and ***P < 0.001 versus control by ANOVA.

After confirming the ability of the mutants to remove FFAs in the GI tract, the anti‐obesity effect of the FFAs‐absorbing mutants was evaluated by feeding the mutant to rats under diet‐induced obesity (DIO) conditions (Fig. 2A). Daily administration of parental strain 3179 or its mutants resulted in successful colonization in the GI tract of the rats after 4 weeks (Table S3). Daily administration of mut1 and mut2 for 22 weeks resulted in consistent reduction in body weight gain (Fig. 2A). At the end, the body weights of uncolonized control, parental 3179, mut1 and mut2 were 514 g (100%), 507 g (101.4%), 435 g (85%) and 424 g (82%), respectively, with maximum difference of 15% for mut1 and 18% for mut2, respectively, compared to the uncolonized control.

In addition to body weight, visceral fat is correlated with obesity as the intake of excess calories in mammals primarily accumulates as visceral fat 29, 30. The visceral fat areas from untreated control, parental strain, mut1 and mut2 were measured 27%, 24%, 14% and 13%, respectively, at 22 weeks using an open‐type 0.3 Tesla MRI (Fig. 2B). These results indicated that the administration of the FFAs‐absorbing *Lactobacillus* mutants could reduce FFAs absorption by the host and thus reduce body weight as well as visceral fat accumulation.

As the body gains weight, it becomes less sensitive to insulin and leptin, which leads to increased plasma concentrations of leptin, insulin and glucose as well as increased LDL and TC 31, 32. The blood profile with respect to the obesity markers was also analyzed at the beginning and end of the feeding experiments. There were significant differences in the lipid profiles within the experimental groups whereas parental 3179 strain failed to show significant differences (Fig. 2C). TG levels of mut1 and mut2 were 63% and 49% of the control respectively. In TC, HDL and LDL, however, only mut2 showed significant difference compared to the uncolonized control. TC levels of mut1 and mut2 were 91% and 74% of the control whereas LDL levels were 104.5% and 58.8% of the control respectively. HDL levels of mut1 and mut2 were 103.1 and 124.9% of the control respectively.

Feeding the rat mut1 and mut2 also significantly reduced the serum insulin levels by 23% and 30%, and serum leptin levels by 30% and 45%, respectively, compared to the uncolonized control (Fig. 2D). The mutant strains also exhibited glucose‐lowering effect, which was expected from their anti‐obesity effect (Fig. 2E). The serum glucose levels were lower in the mut1 and mut2 groups (107.6 mg·dL^−1^ and 108.4 mg·dL^−1^, respectively) than in the uncolonized control and parental strain groups (122.1 mg·dL^−1^ and 123.4 mg·dL^−1^, respectively). Taken together, our FFAs‐absorbing lactobacilli not only inhibited weight gain and body fat accumulation but also improved blood profiles, ameliorating obesity.

### Isolation and characterization of intestinal FFAs‐absorbing *Lactobacillus* JBD301

**Figure 3:**
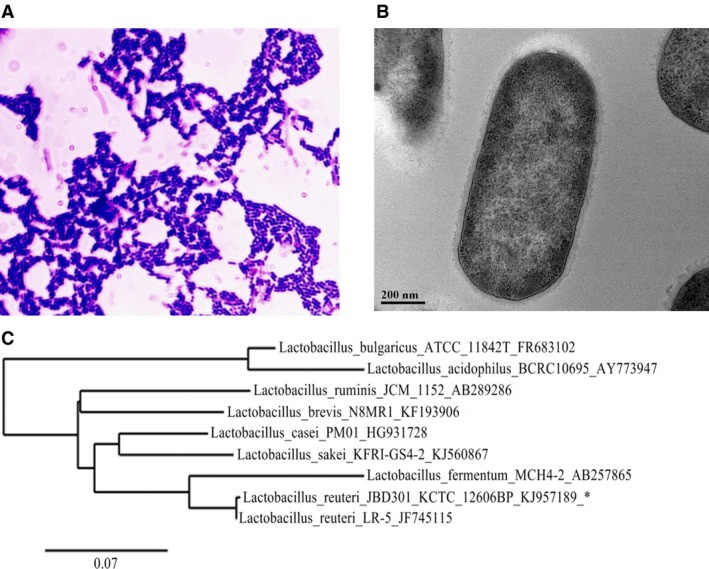
The morphological and molecular characterization of natural FFAs‐absorbing *Lactobacillus* strain, *L. reuteri* JBD301. (A) Gram staining of *Lactobacillus* JBD301. (B) TEM images of *Lactobacillus* JBD301. (C) Phylogenic tree of *Lactobacillus* JBD301. Accession numbers of bacterial strains are marked by an underscore next to the strain number. The phylogenetic tree was constructed by Phylogeny.fr set to build maximum‐likelihood phylogenetic trees (PhyML). The scale bar represents an evolutionary distance.

Because the mutant experiments proved our hypothesis that energy intake can be reduced with the FFAs‐absorbing *Lactobacillus*, we performed high throughput screening to identify intestinal FFAs‐absorbing *Lactobacillus* strains. Fecal samples from lean volunteers were inoculated on MRS agar plates, a selective media for *Lactobacillus*. By screening more than 20 000 strains, we were able to find various *Lactobacillus* strains that absorbed FFAs stronger than the common *Lactobacillus* strains in a FFAs quantitative assay. Animal experiments using the identified FFAs‐absorbing *Lactobacillus* strains in C57BL/6 mice showed that the degree of efficacy on weight gain was positively correlated with the FFAs absorption degree of the *Lactobacillus* strains (Fig. S1). One *Lactobacillus* strain in particular, G5‐1, was shown to absorb FFAs strongly (Fig. S1). Microscopic observations and molecular identification by 16S rDNA sequence analysis identified this strain as an unknown subspecies of *Lactobacillus reuteri* (Fig. 3); thus, it was named *Lactobacillus* JBD301 and deposited into the Korean Collection for Type Cultures (KCTC 12606BP).

### FFAs‐absorbing *Lactobacillus* JBD301 lowered the intestinal FFAs concentrations and thus reduced absorption of FFAs by host whereas increased fecal excretion of FFAs

**Figure 4:**
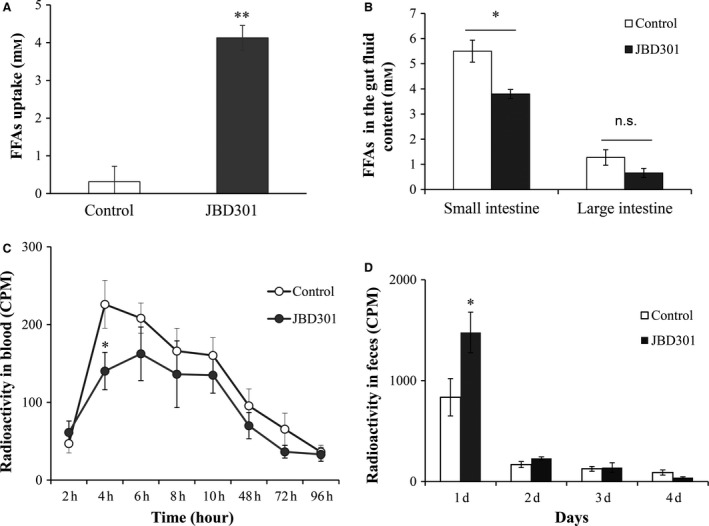
The FFAs‐absorbing *Lactobacillus* JBD301 lowered the intestinal FFAs concentration and limited the absorbable FFAs quantities into host, inhibiting absorption of FFAs by host but promoting excretion of FFAs. (A) The FFAs absorption by *Lactobacillus* JBD301 was compared with common *Lactobacillus* strain by measuring the FFAs concentration in the conditioned media. Values are mean ± SD (*n* = 5). (B) The intestinal FFAs concentrations in the gut fluid of JBD301‐fed host was determined. Seven‐week‐old female C57BL/6 mice were randomized and fed a HFD only (control) or a HFD supplemented daily with 10^7^ CFU of JBD301. After 3 weeks of administration, gut fluid contents were analyzed for total FFAs concentration. Values are mean ± SEM (*n* = 3). (C) The FFAs absorption from dietary fat was determined by measuring the radioactivity in the blood of JBD301‐fed host. Seven‐week‐old female C57BL/6 mice were fed a HFD only (control) or a HFD supplemented daily with 10^7^ CFU of JBD301. After 3 weeks of administration, feed containing radiolabeled fat, ^14^C‐triolein, was administered and radioactivities of the blood samples were analyzed. Values are mean ± SEM (*n* = 7). (D) The FFAs excretion was determined by measuring the radioactivity in the feces of JBD301‐fed host. Seven‐week‐old female C57BL/6 mice were randomized and fed a HFD only (control) or a HFD diet supplemented daily with 10^7^ CFU of JBD301. After 3 weeks of administration, feed containing radiolabeled fat, ^14^C‐triolein, was administered and radioactivities of the feces were analyzed. Values are mean ± SEM (*n* = 7). Statistical significance is shown as *P < 0.05 and **P < 0.01 versus control by ANOVA.

It was shown that FFAs‐absorbing *Lactobacillus* JBD301 was able to absorb FFAs up to 10 times more than other *Lactobacillus* (Fig. 4A, Fig. S1). Next, we investigated whether *Lactobacillus* JBD301 can reduce the absorption of FFAs into host as in the case of the mutant strains. The FFAs absorptions by *Lactobacillus* JBD301 and resulting removals of FFAs from absorbable pool of intestinal FFAs was determined by measuring the FFAs concentration in the gut fluid content where absorption of most FFAs occurs (Fig. 4B). In C57BL/6 mice colonized with *Lactobacillus* JBD301, the FFAs concentrations in fluid contents at small intestine were reduced to 69% of control whereas the FFAs concentrations at large intestine were not much different. The changes in absorption and excretion of dietary fat in the *Lactobacillus* JBD301‐fed host were also determined (Fig. 4C and D). After 3‐week administration of *Lactobacillus* JBD301, radiolabeled dietary fat, ^14^C‐triolein, was orally administered to the mice. The radioactivities from absorbed fat in the blood of *Lactobacillus* JBD301‐fed host were significantly decreased, down to 62% of control at 4 h after intake of radiolabeled food (Fig. 4C). The changes in the amount of excreted dietary fats, which were absorbed into *Lactobacillus* JBD301, were also measured by quantitating the radioactivities in the feces of *Lactobacillus* JBD301‐fed mice (Fig. 4D). Compared to the unfed control, the radioactivities in the feces of JBD301‐fed host were significantly increased, up to 176% of the control after 1 day from radiolabeled food intake.

### FFAs‐absorbing *Lactobacillus* JBD301 inhibited weight gain in host, both in mice and humans

**Figure 5:**
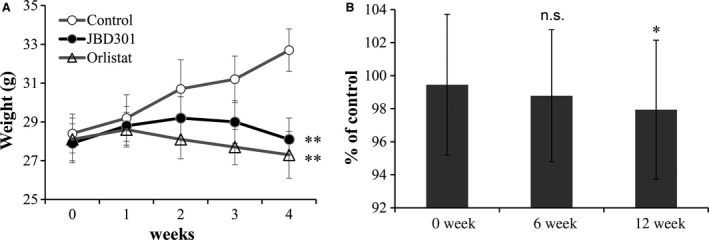
Administration of the FFAs‐absorbing *Lactobacillus* JBD301 significantly reduced body weight of the colonized hosts in both animals and humans. The diet‐induced obese C57BL/6 mice were administered the HFD control diet (○) or the HFD supplemented with *Lactobacillus* JBD301 (●) or orlistat (∆) daily. Seven‐week‐old female C57BL/6 mice were randomized and fed a HFD for 12 weeks to induce obesity. After 12 weeks on the HFD, administration, the animals were randomly divided into groups that received HFD only (control), HFD supplemented with *Lactobacillus* JBD301 (1 × 10^7^ CFU·day^−1^), or HFD supplemented with orlistat (Xenical^®^ at 50 mg·kg^−1^ diet). (A) Body weights were monitored weekly. Values are mean ± SD (*n* = 5). Statistical significance is shown as **P < 0.01 versus control by ANOVA. (B) A random, double‐blind, placebo‐controlled human study was conducted to determine the anti‐obesity efficacy of *Lactobacillus* JBD301 in adults with 25 ≤ BMI < 35. Placebo group (*n* = 19) were administered single capsule of vegetable cream as control while experimental group (*n* = 18) were administered *Lactobacillus* JBD301 at dose of 1 × 10^9^ CFU·day^−1^ for 12 weeks. Values are mean ± SD. Statistical significance of clinical data, body weight at 12 weeks, is shown as *P < 0.05 versus control by Wilcoxon signed rank test (*P* = 0.028).

In accordance with observed inhibition of FFAs absorption, administration of FFAs‐absorbing *Lactobacillus* JBD301 resulted in a significant decrease in body weight of host animal (Fig. 5A). After 4 weeks, the body weight of control groups was 32.7 g with 4.3 g of weight gain. In contrast, JBD301 group was 28.1 g with 0.2 g of weight gain and orlistat group was 27.3 g with 0.8 g of weight loss. The most significant finding in this experiment was that the degree of body weight reduction by *Lactobacillus* JBD301 was as much as that of the mice that had been administered a pharmaceutically effective dose of orlistat.

After confirming that the degree of weight loss by *Lactobacillus* JBD301 could be up to that of a current pharmaceutical drug, a phase 2 clinical trial was conducted to determine the efficacy of *Lactobacillus* JBD301 in obese adults with 25 ≤ BMI < 35 (Fig. S2). This trial was registered with Clinical Research Information Service of Korea as KCT0000452 (https://cris.nih.go.kr). Recruited subjects were included in the trial after screening with inclusion and exclusion criteria. Randomized subjects were daily administered a 450 mg capsule containing either *Lactobacillus* JBD301 at 10^9^ CFU/capsule as experimental ingredient or nondairy creamer as placebo for 12 weeks. In this study, subgroup was analyzed in which 37 subjects received double‐blinded materials which contain either the nondairy creamer as placebo (*n* = 19) or *Lactobacillus* JBD301 as the experimental treatment (*n* = 18) (Fig. S3 and Table S4).

Without any dietary restrictions or additional exercise, changes in the body weight from baseline to endpoint were 0.31% (0.21 kg) in the *Lactobacillus* JBD301 group and 1.77% (1.45 kg) in the placebo group, resulting in a 1.46% (1.24 kg) between‐group difference (Table S5). A Mann–Whitney *U* test showed that there was a statistically significant difference in the percentage change in weight between the *Lactobacillus* JBD301 and the placebo group (*P* = 0.026) as well as in the BMI (*P* = 0.036) from the 0‐week assessment to the 12‐week assessment (Table S5). A Wilcoxon signed rank test also confirmed that there was a statistically significant difference in the pairwise comparison of the percentage of the control between 0 and 12 weeks (*P* = 0.028) (Fig. 5B and Table S6). There were no adverse events related to the treatment.

Because the body weight of humans is heavily affected by food intake and lifestyle, and thus, the body weight of an obese person fluctuates, between‐group differences, *i.e*. weight differences between the control and the experimental group, could be a more important parameter than the value of body weight reduction in obesity clinical trials. In our clinical trial, the weight difference between the placebo and FFAs‐absorbing *Lactobacillus* JBD301 groups was 1.24 kg without calorie restriction, which is far greater than that of the orlistat trial, where 0.71 kg was the weight difference compared to placebo after 180 mg of orlistat for 12 weeks under a hypocaloric diet 33. Therefore, this work shows that the *Lactobacillus* strains with the ability to actively remove intestinal FFAs have anti‐obesity effects as great as the most popular anti‐obesity pharmaceutical drug, orlistat, in animal experiments as well as in a clinical trial.

However, abdominal fat, blood lipid profile, fasting blood glucose, blood insulin,= and HbA1c failed to show significant difference between placebo and experimental group in this trial. Further clinical trials need to consider more numbers of subjects with longer periods of intervention, if possible, with strict control of diet and exercise.

## Discussion

Excess caloric intake from dietary fat is the most important determining factor for obesity, which has become more prevalent throughout the developed world 34, 35. For the vast majority of humans, caloric intake that exceeds caloric expenditure by as little as 1% could result in the accumulation of body fat and eventual obesity 36. Therefore, the 30% reduction in FFAs absorption by orlistat produces significant weight loss 37.

In this work, we demonstrated that the *Lactobacillus* strains that lower intestinal FFAs concentration in the guts show anti‐obesity effects almost as much as the most popular anti‐obesity pharmaceutical drug, orlistat, in animal experiments as well as in a clinical trial (Fig. 5). Although orlistat inhibits FFAs generation for absorption, JBD301 absorbs FFAs and lowers FFA concentration in the gut fluid contents and thus reduces the amount of FFAs available for absorption. Eventually, they both reduce the FFAs uptake into the body and, thus can result in significant weight loss. In addition, *Lactobacillus* JBD301 strains have obvious advantages over the current pharmaceuticals for obesity. The *Lactobacillus* strains do not act on the brain but instead act peripherally and, therefore, have a superior risk‐benefit profile to the current centrally acting drugs. Second, *Lactobacillus* does not act on lipid hydrolysis as orlistat does, which can cause unavoidable side effects in the GI tract, such as anal leakage and oily spotting. Third, *Lactobacillus* is a beneficial probiotic that conveys considerable safety as a drug for long‐term administration.

Despite numerous studies, the direct impact of intestinal microbiota at the genus and species levels on the body weight of host remains unclear until now. Some *Lactobacillus* species are associated with weight gain whereas others are associated with weight loss 38, 39. For instance, administration of *L. acidophillus, L. fermentum* and *L. ingluviei* was associated with weight gain, whereas *L. plantarum* and *L. gasseri* were associated with weight loss. More complicatedly, different strains from the same species often showed different effects on weight 38. Although some strains of *L. reuteri* were associated with obesity, other strains of *L. reuteri* prevent diet‐induced obesity in a strain‐dependent fashion 39, 40. In this study, we showed that lactobacilli either acquired or naturally having the ability to remove FFAs in the GI tract showed anti‐obesity phenotypes, which implies that metabolic activities rather than microbial composition in the intestinal microbiota play determining roles in host phenotypes. These results were further supported by the recent finding that administration of *L. gasseri* SBT2055, a *Lactobacillus* strain having anti‐obesity phenotype, decreased significantly the serum concentration of FFAs in hyperglycemic subject 41. Considering that microbiota seems to affect host obesity by modulating nutrient uptake and energy metabolism, lactobacilli regulating intestinal absorption of FFAs could be the ideal approaches for anti‐obesity phenotypes 42, 43, 44.

Recently, it has been acknowledged that the intestinal microbiota is associated with various human diseases, including obesity, diabetes, metabolic syndrome, inflammatory bowel disease, cognitive functions, cholelithiasis and autism 45, 46, 47. Metagenomic studies demonstrated that the composition of the intestinal microbiota differs in control and disease groups in humans as well as animals 48, 49, 50, 51. However, investigations into the microbial species responsible for diseases have frequently produced conflicting results with no successful attempts to develop a treatment for these diseases by modulating the composition of the intestinal microbiota 52, 53. One possible explanation for the inconsistent correlation between the composition of the intestinal microbiota and the resultant diseases might be due to the intraspecies heterogeneity of bacterial genomes 54. Even within a species, bacterial genomes are highly heterogeneous because of their haploid genomes and the presence of extrachromosomal elements; thus, metabolic activities within a species of bacteria frequently differ from each other 55. To elucidate the determining role of the intestinal microbiota in obesity, based on our results, it is reasonable to investigate the metabolic differences of the intestinal microbiota rather than the compositional differences. We also believe that this approach will lead to the development of living microbial drugs to treat various diseases associated with microbiota.

## Author contributions

S.T.H. and H.J.K. designed research; H.J.C., J.G.Y., I.A.L., M.J.L., Y.F.S., S.P.S., M.A.H.M.J. and J.H.Y. performed research; S.T.H., H.J.K., H.J.C. and J.G.Y. analyzed data; and S.T.H. and H.J.K. wrote the paper.

## Supporting information


**Fig. S1.** Identification of natural *Lactobacillus* strains that strongly absorb FFAs.
**Fig. S2.** Study protocol for randomized controlled trial of effects of *Lactobacillus* JBD301 on obesity.
**Fig. S3.** Flow chart for randomized controlled trial of effects of *Lactobacillus* JBD301on weight loss in obesity.
**Table S1.** The acidification characteristics of milk by *L. acidophilus* KCTC 3179*,* mut1 and mut2.
**Table S2.** Confirmation of colonized *L. acidophilus* KCTC 3179*,* mut1 and mut2 in the GI tract of rats.
**Table S3.** Confirmation of colonized *L. acidophilus* KCTC 3179*,* mut1 and mut2 in the GI tract of the diet‐induced obese rats.
**Table S4.** Study participants' baseline characteristics.
**Table S5.** Comparison of % change in outcome variables at 12th week between the placebo control and the experimental *Lactobacillus* JBD301 groups.
**Table S6.** Comparison of % control in body weight between 0 and 12th week.
**Data S1.** Methods.

## References

[1] Wells JC and Siervo M (2011) Obesity and energy balance: is the tail wagging the dog? Eur J Clin Nutr 65, 1173–1189. 21772313 10.1038/ejcn.2011.132

[2] Visscher TL and Seidell JC (2001) The public health impact of obesity. Annu Rev Public Health 22, 355–375. 11274526 10.1146/annurev.publhealth.22.1.355

[3] Holes‐Lewis KA , Malcolm R and O'Neil PM (2013) Pharmacotherapy of obesity: clinical treatments and considerations. Am J Med Sci 345, 284–288. 23531960 10.1097/MAJ.0b013e31828abcfd

[4] Kang JG and Park CY (2012) Anti‐obesity drugs: a review about their effects and safety. Diabetes Metab J 36, 13–25. 22363917 10.4093/dmj.2012.36.1.13 PMC3283822

[5] Powell AG , Apovian CM and Aronne LJ (2011) New drug targets for the treatment of obesity. Clin Pharmacol Ther 90, 40–51. 21654742 10.1038/clpt.2011.82

[6] Wright SM and Aronne LJ (2011) Obesity in 2010: the future of obesity medicine: where do we go from here? Nat Rev Endocrinol 7, 69–70. 21263435 10.1038/nrendo.2010.231

[7] Poirier P (2011) Weight loss drugs and cardiovascular risks. Curr Cardiovasc Risk Rep 5, 138–144.

[8] James WPT , Caterson ID , Coutinho W , Finer N , Gaal LFV , Maggioni AP , Torp‐Pedersen C , Sharma AM , Shepherd GM , Rode RA *et al* (2010) Effect of sibutramine on cardiovascular outcomes in overweight and obese subjects. N Engl J Med 363, 905–917. 20818901 10.1056/NEJMoa1003114

[9] Yanovski SZ and Yanovski JA (2014) Long‐term drug treatment for obesity: a systematic and clinical review. JAMA 311, 74–86. 24231879 10.1001/jama.2013.281361 PMC3928674

[10] Guercolini R (1997) Mode of action of orlistat. Int J Obes Relat Metab Disord 21, S12–S23. 9225172

[11] Hauptman JB , Jeunet FS and Hartmann D (1992) Initial studies in humans with the novel gastrointestinal lipase inhibitor Ro 18‐0647 (tetrahydrolipstatin). Am J Clin Nutr 55, 309S–313S. 1728845 10.1093/ajcn/55.1.309s

[12] Hill J , Hauptman J , Anderson J , Ken Fujioka K , O'Neil P , Smith D , Zavoral J and Aronne L (1999) Orlistat, a lipase inhibitor, for weight maintenance after conventional dieting: a 1‐y study. Am J Clin Nutr 69, 1108–1116. 10357727 10.1093/ajcn/69.6.1108

[13] Jain R , Chung SM , Jain L , Khurana M , Lau SWJ , Lee JE , Vaidyanathan J , Zadezensky I , Choe S and Sahajwalla CG (2011) Implications of obesity for drug therapy: limitations and challenges. Clin Pharmacol Ther 90, 77–89. 21633345 10.1038/clpt.2011.104

[14] Colman E , Golden J , Roberts M , Egan A , Weaver J and Rosebraugh C (2012) The FDA's assessment of two drugs for chronic weight management. N Engl J Med 367, 1577–1579. 23050510 10.1056/NEJMp1211277

[15] Bays HE (2011) Lorcaserin: drug profile and illustrative model of the regulatory challenges of weight‐loss drug development. Expert Rev Cardiovasc Ther 9, 265–277. 21438803 10.1586/erc.10.22

[16] Gadde KM , Allison DB , Ryan DH , Peterson CA , Troupin B , Schwiers ML and Day WW (2011) Effects of low‐dose, controlled‐release, phentermine plus topiramate combination on weight and associated comorbidities in overweight and obese adults (CONQUER): a randomised, placebo‐controlled, phase 3 trial. Lancet 377, 1341–1352. 21481449 10.1016/S0140-6736(11)60205-5

[17] Halford JC , Boyland EJ , Blundell JE , Kirkham TC and Harrold JA (2010) Pharmacological management of appetite expression in obesity. Nat Rev Endocrinol 6, 255–269. 20234354 10.1038/nrendo.2010.19

[18] Dietrich MO and Horvath TL (2012) Limitations in anti‐obesity drug development: the critical role of hunger‐promoting neurons. Nat Rev Drug Discov 11, 675–691. 22858652 10.1038/nrd3739

[19] Rueda‐Clausen CF , Padwal RS and Sharma AM (2013) New pharmacological approaches for obesity management. Nat Rev Endocrinol 9, 467–478. 23752772 10.1038/nrendo.2013.113

[20] Ley RE , Turnbaugh PJ , Klein S and Gordon JI (2006) Microbial ecology: human gut microbes associated with obesity. Nature 444, 1022–1023. 17183309 10.1038/4441022a

[21] Turnbaugh PJ , Bäckhed F , Fulton L and Gordon JI (2008) Diet‐induced obesity is linked to marked but reversible alterations in the mouse distal gut microbiome. Cell Host Microbe 3, 213–223. 18407065 10.1016/j.chom.2008.02.015 PMC3687783

[22] Ridaura VK , Faith JJ , Rey FE , Cheng J , Duncan AE , Kau AL , Griffin NW , Lombard V , Henrissat B , Bain JR *et al* (2013) Gut microbiota from twins discordant for obesity modulate metabolism in mice. Science 341, 1241214. 24009397 10.1126/science.1241214 PMC3829625

[23] Kadooka Y , Sato M , Imaizumi K , Ogawa A , Ikuyama K , Akai Y , Okano M , Kagoshima M and Tsuchida T (2010) Regulation of abdominal adiposity by probiotics (*Lactobacillus gasseri* SBT2055) in adults with obese tendencies in a randomized controlled trial. Eur J Clin Nutr 64, 636–643. 20216555 10.1038/ejcn.2010.19

[24] Le Barz M , Anhê FF , Varin TV , Desjardins Y , Levy E , Roy D and Marette A (2015) Probiotics as complementary treatment for metabolic disorders. Diabetes Metab J 39, 291–303. 26301190 10.4093/dmj.2015.39.4.291 PMC4543192

[25] Semova I , Carten JD , Stombaugh J , Mackey LC , Knight R , Farber SA and Rawls JF (2012) Microbiota regulate intestinal absorption and metabolism of fatty acids in the zebrafish. Cell Host Microbe 12, 277–288. 22980325 10.1016/j.chom.2012.08.003 PMC3517662

[26] Lin WH , Hwang CF , Chen LW and Tsen HY (2006) Viable counts, characteristic evaluation for commercial lactic acid bacteria products. Food Microbiol 23, 74–81. 16942989 10.1016/j.fm.2005.01.013

[27] Johansson ML , Molin G , Jeppsson B , Nobaek S , Ahrné S and Bengmark S (1993) Administration of different *Lactobacillus* strains in fermented oatmeal soup: in vivo colonization of human intestinal mucosa and effect on the indigenous flora. Appl Environ Microbiol 59, 15–20. 8439146 10.1128/aem.59.1.15-20.1993 PMC202048

[28] Baek SH , Chung HJ , Lee HK , D'Souza R , Jeon Y , Kim HJ , Kweon SJ and Hong ST (2014) Treatment of obesity with the resveratrol‐enriched rice DJ‐526. Sci Rep 4, 3879. 24464364 10.1038/srep03879 PMC3902431

[29] Leenen R , van der Kooy K , Deurenberg P , Seidell JC , Weststrate JA , Schouten FJ and Hautvast JG (1992) Visceral fat accumulation in obese subjects: relation to energy expenditure and response to weight loss. Am J Physiol 263, E913–E919. 1443124 10.1152/ajpendo.1992.263.5.E913

[30] Keno Y , Matsuzawa Y , Tokunaga K , Fujioka S , Kawamoto T , Kobatake T and Tarui S (1991) High sucrose diet increases visceral fat accumulation in VMH‐lesioned obese rats. Int J Obes 15, 205–211. 2045213

[31] Després JP and Lemieux I (2006) Abdominal obesity and metabolic syndrome. Nature 444, 881–887. 17167477 10.1038/nature05488

[32] Molavi B , Rasouli N and Kern PA (2006) The prevention and treatment of metabolic syndrome and high‐risk obesity. Curr Opin Cardiol 21, 479–485. 16900012 10.1097/01.hco.0000240586.76344.f5

[33] Drent ML , Larsson I , William‐Olsson T , Quaade F , Czubayko F , von Bergmann K , Strobel W , Sjöström L and van der Veen EA (1995) Orlistat (RO 18‐0647), a lipase inhibitor, in the treatment of human obesity: a multiple dose study. Int J Obes Relat Metab Disord 19, 221–226. 7627244

[34] Rand CS (1987) Obesity and caloric intake. J Chronic Dis 40, 911–912. 3597693 10.1016/0021-9681(87)90194-9

[35] National Institutes of Health (NIH), National Heart, Lung, and Blood Institute (NHLBI) (1998) Clinical Guidelines on the Identification, Evaluation, and Treatment of Overweight and Obesity: the Evidence Report. US Government Press, Washington, DC, USA.

[36] Hill JO (2006) Understanding and addressing the epidemic of obesity: an energy balance perspective. Endocr Rev 27, 750–761. 17122359 10.1210/er.2006-0032

[37] Davidson MH , Hauptman J , DiGirolamo M , Foreyt JP , Halsted CH , Heber D , Heimburger DC , Lucas CP , Robbins DC , Chung J *et al* (1999) Weight control and risk factor reduction in obese subjects treated for 2 years with orlistat: a randomized controlled trial. JAMA 281, 235–242. 9918478 10.1001/jama.281.3.235

[38] Million M , Angelakisa E , Paulb M , Armougoma F , Leibovicic L and Raoulta D (2012) Comparative meta‐analysis of the effect of *Lactobacillus* species on weight gain in humans and animals. Microb Pathog 53, 100–108. 22634320 10.1016/j.micpath.2012.05.007

[39] Fak F and Backhed F (2012) *Lactobacillus reuteri* prevents diet‐induced obesity, but not atherosclerosis, in a strain dependent fashion in Apoe‐/‐ mice. PLoS One 7, e46837. 23056479 10.1371/journal.pone.0046837 PMC3467285

[40] Qiao Y , Sun J , Xia S , Li L , Li Y , Wang P , Shi Y and Le G (2015) Effects of different *Lactobacillus reuteri* on inflammatory and fat storage in high‐fat diet‐induced obesity mice model. J Funct Foods 14, 424–434.

[41] Ogawa A , Kadooka Y , Kato K , Shirouchi B and Sato M (2014) *Lactobacillus gasseri* SBT2055 reduces postprandial and fasting serum non‐esterified fatty acid levels in Japanese hypertriacylglycerolemic subjects. Lipids Health Dis 13, 36. 24548293 10.1186/1476-511X-13-36 PMC3944925

[42] Caesar R , Fåk F and Bäckhed F (2010) Effects of gut microbiota on obesity and atherosclerosis via modulation of inflammation and lipid metabolism. J Intern Med 268, 320–328. 21050286 10.1111/j.1365-2796.2010.02270.x

[43] Krajmalnik‐Brown R , Ilhan ZE , Kang DW and DiBaise JK (2012) Effects of gut microbes on nutrient absorption and energy regulation. Nutr Clin Pract 27, 201–214. 22367888 10.1177/0884533611436116 PMC3601187

[44] Collins SM and Bercik P (2009) The relationship between intestinal microbiota and the central nervous system in normal gastrointestinal function and disease. Gastroenterology 136, 2003–2014. 19457424 10.1053/j.gastro.2009.01.075

[45] Tilg H and Kaser A (2011) Gut microbiome, obesity, and metabolic dysfunction. J Clin Invest 121, 2126–2132. 21633181 10.1172/JCI58109 PMC3104783

[46] Sekirov I , Russell SL , Antunes LC and Finlay BB (2010) Gut microbiota in health and disease. Physiol Rev 90, 859–904. 20664075 10.1152/physrev.00045.2009

[47] DiBaise JK , Frank DN and Mathur R (2012) Impact of the gut microbiota on the development of obesity: current concepts. Am J Gastroenterol Suppl 1, 22–27.

[48] Qin J , Li Y , Cai Z , Li S , Zhu J , Zhang F , Liang S , Zhang W , Guan Y , Shen D *et al* (2012) A metagenome‐wide association study of gut microbiota in type 2 diabetes. Nature 490, 55–60. 23023125 10.1038/nature11450

[49] Turnbaugh PJ , Ley RE , Mahowald MA , Magrini V , Mardis ER and Gordon JI (2006) An obesity‐associated gut microbiome with increased capacity for energy harvest. Nature 444, 1027–1031. 17183312 10.1038/nature05414

[50] Turnbaugh PJ , Ley RE , Hamady M , Fraser‐Liggett C , Knight R and Gordon JI (2007) The human microbiome project: exploring the microbial part of ourselves in a changing world. Nature 449, 804–810. 17943116 10.1038/nature06244 PMC3709439

[51] Bäckhed F , Fraser CM , Ringel Y , Sanders ME , Sartor RB , Sherman PM , Versalovic J , Young V and Finlay BB (2012) Defining a healthy human gut microbiome: current concepts, future directions, and clinical applications. Cell Host Microbe 12, 611–620. 23159051 10.1016/j.chom.2012.10.012

[52] Clemente JC , Ursell LK , Parfrey LW and Knight R (2012) The impact of the gut microbiota on human health: an integrative view. Cell 148, 1258–1270. 22424233 10.1016/j.cell.2012.01.035 PMC5050011

[53] Delzenne NM , Neyrinck AM , Bäckhed F and Cani PD (2011) Targeting gut microbiota in obesity: effects of prebiotics and probiotics. Nat Rev Endocrinol 7, 639–646. 21826100 10.1038/nrendo.2011.126

[54] Musso G , Gambino R and Cassader M (2011) Interactions between gut microbiota and host metabolism predisposing to obesity and diabetes. Ann Rev Med 62, 361–380. 21226616 10.1146/annurev-med-012510-175505

[55] Oliverio AM and Katz LA (2014) The dynamic nature of genomes across the tree of life. Genome Biol Evol 6, 482–488. 24500971 10.1093/gbe/evu024 PMC3971579

