# Supplementary material for: Intestinal removal of free fatty acids from hosts by Lactobacilli for the treatment of obesity
Source: FEBS Open Bio. 2016 Jan 18;6(1):64–76. doi: 10.1002/2211-5463.12024 (PMC4794792; doi:10.1002/2211-5463.12024)
Supplement: Supplementary file 1 — Fig. S1. Identification of natural Lactobacillus strains that strongly absorb FFAs. Fig. S2. Study protocol for randomized controlled trial of effects of Lactobacillus JBD301 on obesity. Fig. S3. Flow chart for randomized controlled trial of effects of Lactobacillus JBD301on weight loss in obesity. Table S1. The acidification characteristics of milk by L. acidophilus KCTC 3179, mut1 and mut2. Table S2. Confirmation of colonized L. acidophilus KCTC 3179, mut1 and mut2 in the GI tract of rats. Table S3. Confirmation of colonized L. acidophilus KCTC 3179, mut1 and mut2 in the GI tract of the diet‐induced obese rats. Table S4. Study participants' baseline characteristics. Table S5. Comparison of % change in outcome variables at 12th week between the placebo control and the experimental Lactobacillus JBD301 groups. Table S6. Comparison of % control in body weight between 0 and 12th week. Data S1. Methods. [file FEB4-6-64-s001.docx]

**Intestinal removal of free fatty acids from hosts by *Lactobacilli* for the treatment of obesity**

Hea-Jong Chung,^a,1^ Jae Gak Yu,^b,1^ In-Ah Lee,^b^ Ming-Jie Liu,^a^ Yan-Fei Shen,^a^ Satya Priya Sharma,^a^ Mohammad Abu Hena Mostofa Jamal,^a^ Jun-Hyun Yoo,^c^ Hyeon-Jin Kim,^b,*^ Seong-Tshool Hong^a,*^

^a^Department of Biomedical Sciences and Institute for Medical Science, Chonbuk National University Medical School, Jeonju, Chonbuk, 561-180, Korea

^b^JINIS BDRD institute, JINIS Biopharmaceuticals Co., Wanju, Chonbuk, 565-902, Korea

^c^Department of Family Medicine, Samsung Medical Center, Sungkyunkwan University School of Medicine, Seoul, 135-710, Korea.

^*^ Corresponding authors at: Department of Biomedical Sciences and Institute for Medical Science, Chonbuk National University Medical School, Keumam-Dong San 2-20, Jeonju, Chonbuk, 561-180, South Korea. Tel.: +82632703105; fax: +82632703105. E-mail address: [seonghong@JBNU.ac.kr](mailto:seonghong@JBNU.ac.kr) (S.-T. Hong), JINIS BDRD institute, JINIS Biopharmaceuticals Co., 913 Kwahak-Ro, Bongdong, Wanju, Chonbuk, 565-902, South Korea. Tel.: +82632143935; fax: +82632143934. E-mail address: [hjkim@jinisbio.com](mailto:hjkim@jinisbio.com) (H.-J. Kim),

^1^ These authors contributed equally to this work.

**Supplementary Figures**

**
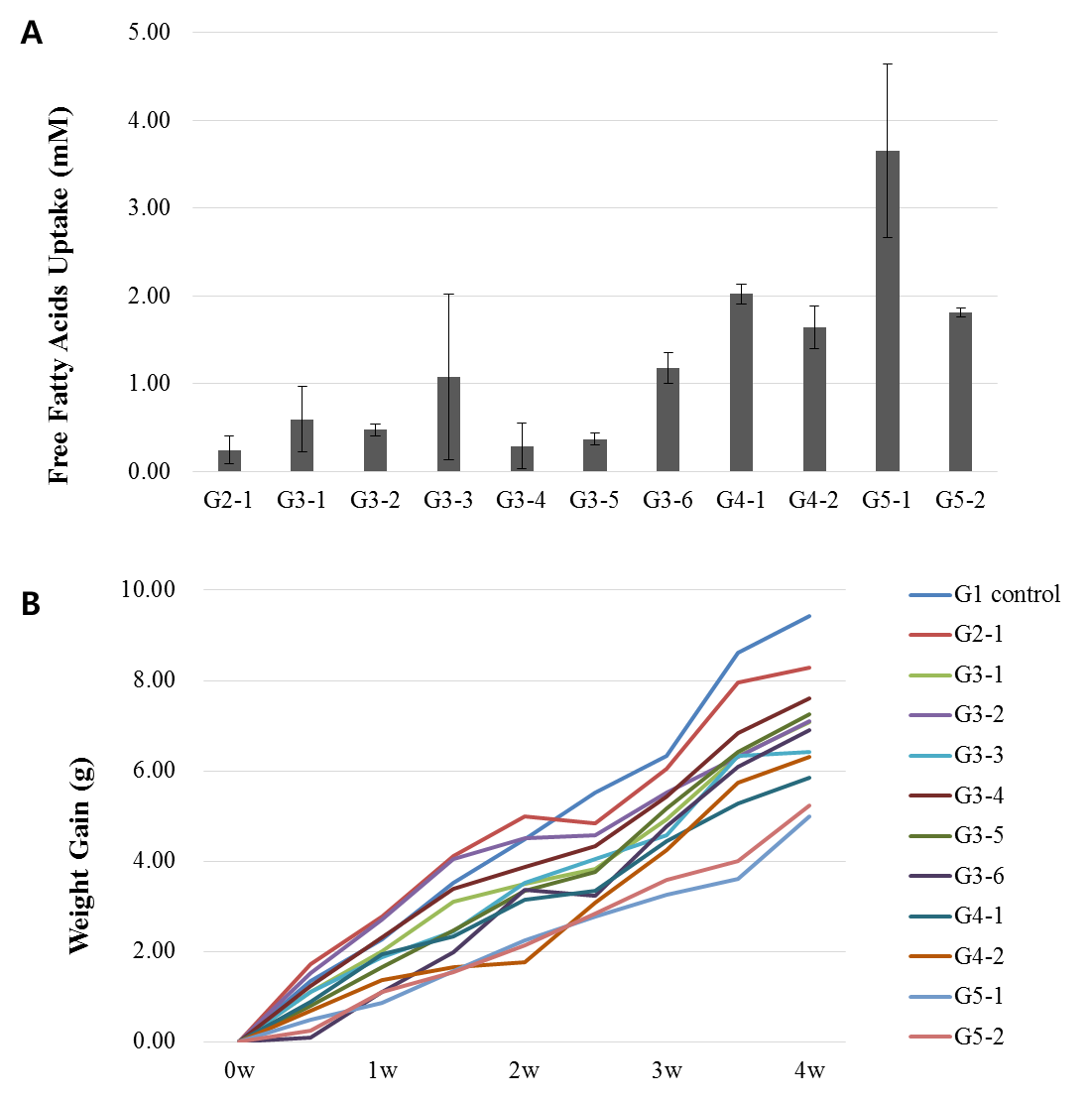
**

**Supplementary Figure S1.** Identification of natural *Lactobacillus* strains that strongly absorb FFAs. (A) FFAs absorption of *Lactobacillus* strains was determined by measuring the FFAs concentration in the conditioned media of the *Lactobacilli* culture. Values are mean ± SD (n = 5 per group). (B) Inhibition of body weight gain by administration of the identified FFAs-removing *Lactobacillus* strains. Three-month-old C57BL/6 female mice were randomized and fed a high fat diet only (control) or a diet supplemented with each *Lactobacilli* for 4 weeks. Body weight was monitored twice per week.


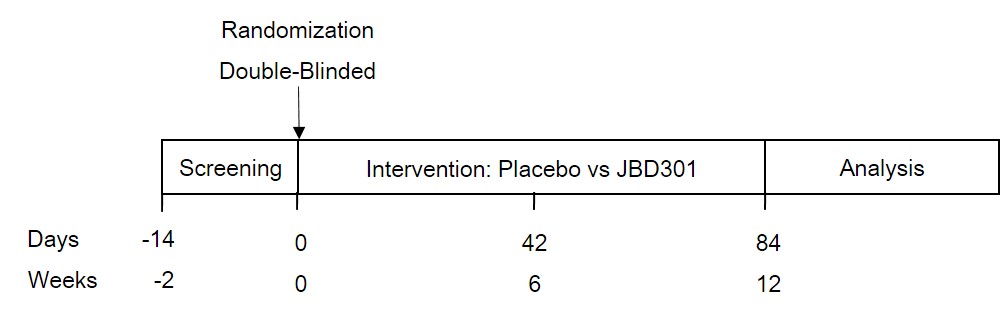


**Supplementary Figure S2.** Study protocol for randomized controlled trial of effects of *Lactobacillus* JBD301 on obesity.

**
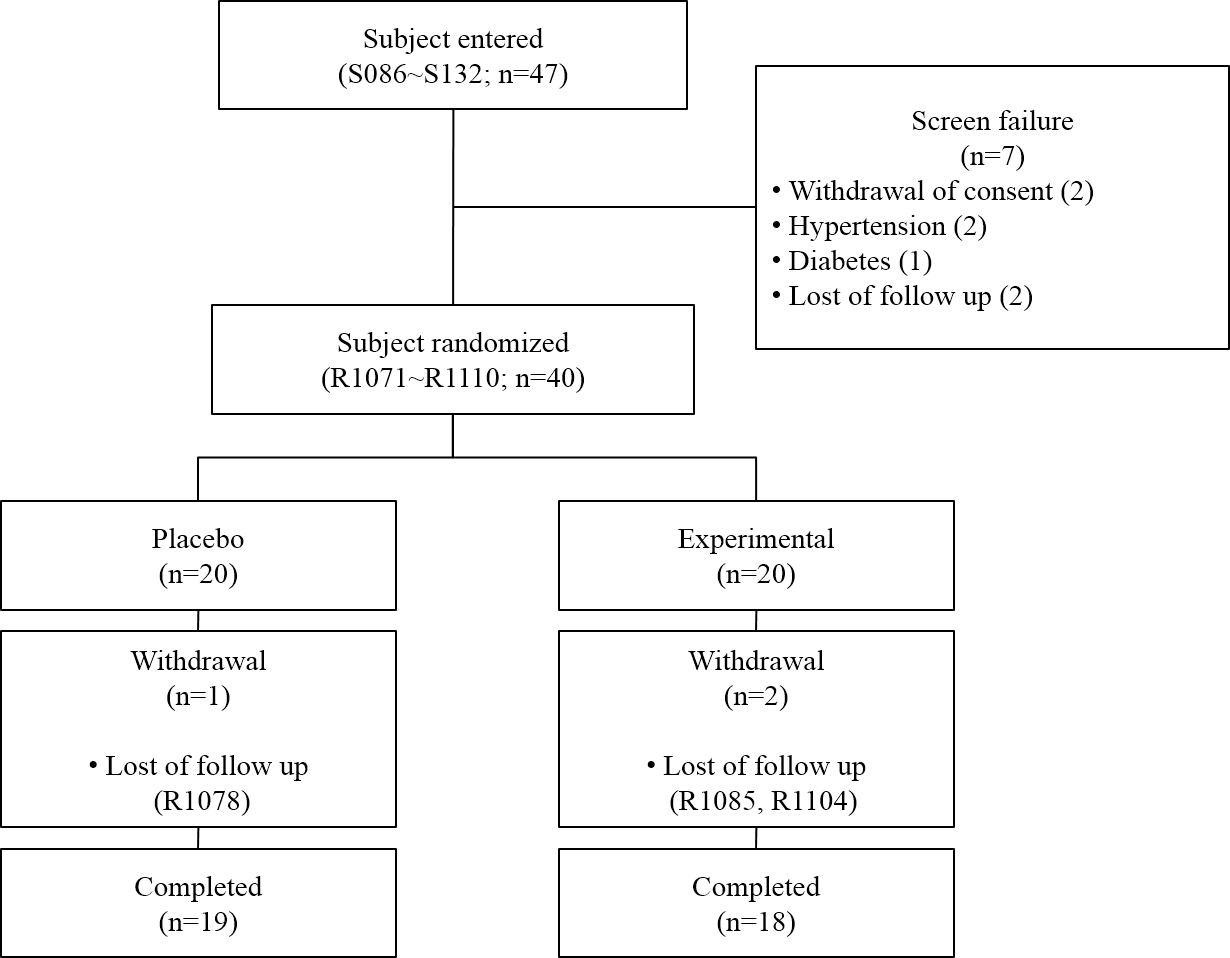
**

**Supplementary Figure S3.** Flow chart for randomized controlled trial of effects of *Lactobacillus* JBD301on weight loss in obesity.

**Supplementary Tables**

**Supplementary Table S1.** The acidification characteristics of milk by *L. acidophilus* KCTC 3179*,* mut1 and mut2

|  | pH value of yogurt | | |
| --- | --- | --- | --- |
|  | **24 hours** | **48 hours** | **72 hours** |
| 3179 | 6.78 ± 0.16 | 3.82 ± 0.24 | 3.66 ± 0.13 |
| mut1 | 7.12 ± 0.16 | 4.42 ± 0.05 | 3.97 ± 0.12 |
| mut2 | 6.59 ± 0.24 | 3.77 ± 0.14 | 3.69 ± 0.12 |

NOTE. Each *Lactobacillus* cell was inoculated into the sterile reconstituted skim milk supplemented with 2% glucose to make yogurt. The pH values of the each yogurt were determined at the indicated time points (24 hours, 48 hours and 72 hours) to measure their acidification activities. Values are mean ± SEM of 10 samples each.

**Supplementary Table S2.** Confirmation of colonized *L. acidophilus* KCTC 3179*,* mut1 and mut2 in the GI tract of rats

| **Strain** | Log_10_ [CFU of *Lactobacilli*/g (wet weight) in organ] | |
| --- | --- | --- |
|  | **In Stomach** | **In Small intestine** |
| Control | Non-detectable | 6.64 ± 0.22 |
| 3179 | 8.54 ± 0.20 | 8.64 ± 0.14 |
| mut1 | 8.61 ± 0.19 | 8.50 ± 0.19 |
| mut2 | 8.50 ± 0.22 | 8.63 ± 0.23 |

NOTE. After feeding rats yogurt fermented with *L. acidophilus* KCTC 3179, mut1 and mut2 for 8 weeks each, stomach and small intestine samples were taken from each group to count the colonies forming units (CFU). Control means rats that were not fed yogurt. Values are mean ± SEM of 5 samples each.

**Supplementary Table S3.** Confirmation of colonized *L. acidophilus* KCTC 3179*,* mut1 and mut2 in the GI tract of the diet-induced obese rats

| **Strain** | Log_10_ [CFU of *Lactobacilli/*g (wet weight) in organ] | |
| --- | --- | --- |
|  | **In Stomach** | **In Small intestine** |
| Control | Non-detectable | 5.98 ± 0.21 |
| 3179 | 8.15 ± 0.29 | 9.14 ± 0.24 |
| mut1 | 8.32 ± 0.23 | 8.89 ± 0.22 |
| mut2 | 8.19 ± 0.17 | 9.05 ± 0.19 |

NOTE. After feeding rats HFD with yogurt fermented with *L. acidophilus* KCTC 3179, mut1 and mut2 for 4 weeks each, stomach and small intestine samples were taken from each group to count the colonies forming units (CFU). Control means rats that were not fed yogurt. Values are mean ± SEM of 5 samples each.

S**upplementary Table S4.** Study participants’ baseline characteristics

|  | **Group** | |  |
| --- | --- | --- | --- |
| Variable | ***Lactobacillus* JBD301** | **Control** | ***P* value** |
| All participants | 18 (48.6) | 19 (51.4) |  |
| Sex, male | 7 (38.9) | 13 (68.4) | 0.103 |
| Sex, female | 11 (61.1) | 6 (31.6) |  |
| Age mean ± SEM | 39.4 ± 2.7 | 42.1 ± 3.1 | 0.578 |
| Height mean ± SEM | 165.0 ± 2.4 | 169.0 ± 2.3 | 0.233 |
| Weight mean ± SEM | 79.6 ± 3.4 | 80.1 ± 3.0 | 0.822 |
| BMI mean ± SEM | 29.0 ± 0.7 | 27.9 ± 0.5 | 0.150 |

NOTE. Values are n (%) for categorical variables and mean ± SEM for continuous variables. *P* values were derived from Fisher’s exact test for categorical data and the Mann-Whitney U test for continuous data.

**Supplementary Table S5.** Comparison of % change in outcome variables at 12^th^ week between the placebo control and the experimental *Lactobacillus* JBD301 groups

(Unit: %)

|  | ***Lactobacillus*** JBD301 | | Control | |  |
| --- | --- | --- | --- | --- | --- |
| % change in Variable | n | Mean ± SEM | n | Mean ± SEM | *P* value |
| Total abdominal Fat area (cm^2^) | 18 | 1.64 ± 4.26 | 19 | 3.45 ± 4.23 | 1.000 |
| Body fat mass (g) | 18 | -0.38 ± 2.09 | 19 | 3.41 ± 1.17 | 0.053 |
| Weight (kg) | 18 | 0.31 ± 0.71 | 19 | 1.77 ± 0.40 | 0.026* |
| BMI (kg/m) | 18 | 0.32 ± 0.76 | 19 | 1.65 ± 0.46 | 0.036* |
| Hip circumference (cm) | 18 | -0.20 ± 0.45 | 19 | 0.76 ± 0.31 | 0.126 |
| HbA1c (%) | 18 | -1.61 ± 1.02 | 19 | 1.39 ± 1.16 | 0.118 |

NOTE. **P* values were derived from the Mann-Whitney U test and considered significant if *p* < 0.05.

**Supplementary Table S6.** Comparison of % control in body weight between 0 and 12^th^ week

(Unit: %)

|  | **0 week** | **12 week** | ***P* value** |
| --- | --- | --- | --- |
| % control in body weight | 99.45 ± 4.26 | 97.94 ± 4.21 | 0.028* |

NOTE. * *P* values were derived from the Wilcoxon signed rank test and considered significant if *p* < 0.05. Values are mean ± SEM.

**Supplementary Methods**

*2.1. Animal experiments*

All animals were maintained under a 12-h light / 12-h dark cycle throughout the experiment. The temperature was a constant 22 ± 1 °C and with 40−50% humidity. After 1 week of familiarization in this environment, the animals were randomly divided into control or experimental groups for each set of experiments.

All animal care and use were performed strictly in accordance with the ethical guidelines by the Ethics Committee of Chonbuk National University Laboratory Animal Center, and the animal study protocol was approved by the institution (CBU No. 2012-0040).

*2.2. Isolation of FFAs-absorbing Lactobacillus mutants*

L. acidophilus KCTC 3179 cells were grown statically in MRS medium, pH 7.2, at 37 °C in a BBL Gas-Pack for anaerobic culture, unless otherwise noted. Chemical mutagenesis of L. acidophilus KCTC 3179 was performed as described below to obtain the mut1 mutant. N-methyl-N-nitro-N-nitrosoguanidine was added to a final concentration of 2 mg/ml into a 24 h culture of L. acidophilus KCTC 3179 cells in MRS broth. After shaking at 25 °C for 30 min to achieve over 99% mortality *of* L. acidophilus under mutagen treatment, the *L. acidophilus* cells were washed three times with fresh MRS broth and resuspended in fresh MRS broth. After serial dilution, the treated cells were spread on MRS agar plates and incubated at 37 °C under anaerobic conditions. After 48 h, the mutant colonies were transferred into separate wells of a 384-well plate containing 50 µL of MRS broth. The inoculated colonies were cultured under anaerobic conditions at 37 °C without shaking. Following overnight incubation, the plate was replicated using a 384-pin replicator into two new plates, one with fresh MRS broth and another with the same broth containing 0.1 nCi/mL of ^14^C-palmitic acid. Then, the plates were wrapped with parafilm and incubated at 37 °C for 30 min with gentle shaking. After incubation, 2 µL of the ^14^C-labeled culture in each well was transferred with a 384-pin replicator onto nylon membranes. After drying the membrane with the semi-dry system, the free ^14^C-palmitic acid in the bacterial spots on the membrane was removed by washing three times with MRS broth. The washed membrane was exposed to X-ray film at −80 °C for 3 days. After development, the X-ray film was scanned, and the relative dot density was analyzed with Gel-Pro analyzer software. The high-dot-density colonies were selected, and the strain absorbing FFAs to strongest was identified and named mut1. After obtaining mut1 from NTG mutagenesis, 4-nitroquinoline 1-oxide was used to mutagenize mut1 by the mutagenesis method described above to generate the 2^nd^-round mutant, mut2.

*2.3. In vitro characterization of the L. acidophilus mutants*

To evaluate the *in vitro* FFAs absorption of the *Lactobacillus* strains, the radioactivity was measured after incubation with ^14^C-labeled palmitic acid. The mutant strains were inoculated into 2 mL of MRS broth and incubated. At the end of the exponential growth phase, the cell density was estimated by measuring the absorbance at 600 nm. The cells were harvested by centrifugation and resuspended into fresh MRS broth containing 1 nCi/mL of ^14^C-palmitic acid. After incubation for 30 min at 37 °C, the ^14^C-labeled cells were washed 3 times with MRS broth. After resuspension in 1 mL of fresh MRS broth, 200 μL of the cell suspension was carefully transferred into a 4-mL scintillation vial containing 2 mL of liquid scintillation cocktail (PerkinElmer Life Sciences). The mixture was vigorously vortexed for 1 min, and then the ^14^C activity was determined by liquid scintillation spectrophotometry. To test acid production, the *Lactobacillus* mutants were cultured at 37 °C in MRS broth until the end of the exponential growth phase. The cell cultures (1 mL each) were inoculated into bottles containing 100 mL of sterile reconstituted skim milk (10%) and glucose (2%). The pH changes of the fermented milk were determined after incubation for 24, 48 and 72 h. For cell growth measurement, 5 ml of the resulting yogurt after 48 h of culture was transferred to a 15-mL conical tube and vortexed vigorously. Then, 1 mL of the homogenized sample was serially diluted with sterile PBS, and 50 µL from each dilution was plated on a MRS plate. The plates were cultured under anaerobic conditions for 48 h to count the visible colonies.

*2.4. Colonization of Lactobacillus in the gastrointestinal tract of administered host*

Male Sprague-Dawley (SD) rats (Dae Han Biolink, Korea) weighing 200−220 g were housed two per cage and provided normal rat food and water *ad libitum* during the first week. The normal diet used in this study was standard rat food (60% complex carbohydrates, 22% protein, 3.5% fat, 5% fiber, 8% crude ash, 0.6% calcium, and 1.2% phosphorus). After 1 week of familiarization in this environment, the rats were fed tested *Lactobacillus* at 10^7^ CFU for 8 weeks for colonization. The yogurt used for feeding was fermented with 10% non-fat milk, 2% sugar, and 1% *L. acidophilus* culture.

After 8 weeks of *Lactobacillus* feeding for colonization, rats from each of the groups were randomly selected and killed under anesthesia with ether. The gastrointestinal organs, stomach and small intestine, were collected immediately and weighed. The samples were transferred into 50-mL conical tubes and diluted with sterile saline to yield a 10-fold dilution (wt/vol). Then, the samples in saline were homogenized with a homogenizer to release the contents of the gastrointestinal organs. After serial dilution, the homogenates were plated on MRS agar plates and incubated anaerobically for 48 h. The viable *Lactobacillus* numbers were assessed by counting the colonies formed on the MRS agar plates and expressed as log_10_ CFU per gram (wet weight) of various regions of the gastrointestinal organs.

*2.5. Blood and fecal analysis of FFAs in hosts fed FFAs-absorbing mutants*

Male Sprague-Dawley (SD) rats (Dae Han Biolink) weighing 200−220 g were housed two per cage and provided normal rat food and water *ad libitum* during the first week. After 1 week of familiarization in this environment, the rats were fed standard diet (60% complex carbohydrates, 22% protein, 3.5% fat, 5% fiber, 8% crude ash, 0.6% calcium, and 1.2% phosphorus) supplemented with tested *Lactobacillus* 10^7^ CFU daily. The yogurt used for supplementation was fermented with 10% non-fat milk, 2% sugar, and 1% *L. acidophilus* culture. After 8 weeks for colonization, radiolabeled fat was orally administered. The long-chain triglyceride triolein was used as the substrate in this experiment. Approximately 1 µCi of ^14^C-labeled triolein was added per ml of unlabeled triolein, and the solvent was evaporated at RT under nitrogen overnight. The triolein mixture was administered at 0.5 mmol/100 g of body weight to the *Lactobacillus*-colonized SD rats. Blood samples were collected by cardiac puncture at 120, 240, 360, 480 and 600 min. Each serum sample (100 μL) was mixed immediately with 1.8 mL of liquid scintillation cocktail, and the ^14^C activity was determined by liquid scintillation spectrophotometry. FFAs in the fecal fluid contents were also quantified. The total dry feces were dissolved in 20% NaCl in 1 N HCl and homogenized briefly. After centrifugation of the fecal mixture at 25,000 rpm for 20 min, the supernatants were collected. Ethyl acetate (HPLC grade) was added to the supernatants, mixed, and centrifuged at 16,000 × g for 5 min. The ethyl acetate layers of the centrifuged mixtures were collected and evaporated by nitrogen gas. Each dried samples were derivatized with 100 μL of N, O-bis(trimethylsilyl) trifluoroacetamide containing 1% trimethylchlorosilane (Sigma-Aldrich, St. Louis, MO, USA) by incubation at 80 °C for 30 min. After the derivatization reaction was complete, 1 μL of the resulting reactants was subjected to GC/MS. The GC/MS analysis was performed using a GC/MS-QP2010 Ultra (Shimadzu, Kyoto, Japan) with a fused silica capillary column (DB-5MS, Agilent, Santa Clara, CA, USA), and the data were acquired using the Lab Solution software (Shimadzu). The initial column temperature was 70 °C for 2 min, followed by a 10 °C/min ramp to 300 °C for 5 min. The flow rate of helium gas through the column was 1 ml/min. The transfer line and ion-source temperatures were 250 °C and 200 °C, respectively. A scan speed of two thousand was recorded over the mass range of 40−500 m/z using the Advanced Scanning Speed Protocol (Shimadzu). The identification of FFAs was based on the retention time of standard compounds and was assisted by the NIST 08 and Wiley libraries.

*2.6. Effect of FFAs-absorbing mutants in diet-induced obese rats*

Three-month-old male SD rats (Daehan Biolink) weighing 200−220 g were housed two per cage and provided normal rat food and water *ad libitum* during the first week. After 1 week of familiarization in this environment, the rats were randomized and fed high-fat diet (HFD) only or a HFD diet supplemented with tested *Lactobacillus* (*L. acidophilus* KCTC3179, mut1 or mut2). The HFD was made with standard rat food plus 20% pig lard, which changes the composition to 48% complex carbohydrates, 17.6% protein, 22.8% fat, 4% fiber, 6.4% crude ash, 0.48% calcium, and 0.96% phosphorus. The yogurt used for feeding was fermented with 10% non-fat milk, 2% sugar, and 1% *L. acidophilus* culture for daily administration of 10^7^ CFU. The body weights of the SD rats were measured weekly for 22 weeks. At the end of the experiments, magnetic resonance imaging (MRI) analysis was performed to measure the visceral (or abdominal) fat with a Bruker Biospec 47/40 4.7-Tesla instrument. The rats were anesthetized with a combination of zoletil (25 mg/kg) and rompun (10 mg/kg). To obtain the images, the rats were placed in a prone position in the magnet. The MRIs were recorded using the body coil as the transmitter and receiver. A series of T1-weighted transaxial scans for the measurement of visceral and subcutaneous fat were acquired from a region extending from 8 cm above to 8 cm below the 4^th^ and 5^th^ lumbar interspace, and the fat areas were measured using the Image J program. Blood samples from the experimental rats were collected at 0 and 22 weeks to determinate the serum biochemical values. All animals were fasted overnight prior to blood collection. The whole blood samples from the rats were collected by cardiac puncture under anesthesia with ether. After centrifugation at 2,000 × g for 10 min at 4 °C, the serum samples were aliquoted and stored at −70 °C until analysis. The sera were analyzed for biochemical characteristics using commercially available kits. The serum TC, HDL and, LDL levels as well as the TG concentrations were detected with the ELISA kits described in the Reagents section above.

*2.7. Screening for intestinal FFAs-absorbing Lactobacillus strains*

*Lactobacillus* strains were isolated from the feces of healthy lean adult volunteers. Fresh fecal samples (stools) were appropriately diluted with MRS broth and spread onto MRS agar plates for incubation in anaerobic jars at 37 °C. After 48 h of incubation, the *Lactobacillus* colonies grown on the MRS agar plates were transferred into a 96-well plate containing MRS broth, and the plates of these isolates were incubated anaerobically at 37 °C. Using an automated liquid handler (Beckman, Brea, CA, USA), these plates were replicated into new 96-well plates for maintenance and screening purposes. For high throughput screening of the *Lactobacillus* strains that had natural FFAs-absorbing capability, 10 μL of the individual *Lactobacilli* overnight cultures in the 96-well plates were inoculated in 250 μL of MRS broth containing 0.5% (w/v) Brij™ 58 and 0.25 mM sodium palmitate. After 15 h of incubation at 37 °C under anaerobic conditions, the supernatants were collected by centrifugation of the 96-well plates at 4,000 rpm for 10 min at 4 °C. The quantities of FFAs in the cultured media were determined by reading the optical density at 570 nm using the EnzyChrom™ free fatty acid assay kit (Bio-Assay Systems) as described by the manufacturer. Using the concentration of FFAs in the media, the quantity of FFAs uptake from the media was calculated.

*2.8. Identification of intestinal FFAs-absorbing Lactobacillus JBD301*

The *Lactobacillus* strain with the strongest FFAs-absorbing ability, G5-1, was selected among the identified intestinal strains by the aforementioned high throughput screening. This strain was identified as a new subspecies of *L. reuteri* following taxonomic analyses and was named *Lactobacillus* JBD301. The taxonomic classification and phylogenetic analysis of the G5-1 strain was based on the 16S rDNA gene and morphological observations. For morphological analysis, freshly cultured *Lactobacillus* was used for standard light microscopy after Gram staining as well as Transmission Electron Microscopy (TEM) (H7650) using the ES1000W device [1]. For molecular analysis, DNA was extracted from a JBD301 culture according to the DNA extraction kit protocol (Nucleospin Tissue MN ref: 740952.250). For amplification of the 16s rDNA, universal primers were selected: 27 F (5^΄^-AGAGTTTGATCCTGGCTCAG-3΄) as the forward primer and 1492R (5^΄^-GGTTACCTTGTTACGACTT-3΄) as the reverse primer. PCR was performed in an automated thermal cycler with an initial denaturation at 95 ºC for 5 min, followed by 30 cycles at 95 ºC for 30 sec, 52 ºC for 45 sec, 72 ºC for 2 min, and then 72 ºC for 10 min. Following the deposition of the obtained 16s rDNA sequence into the NCBI GenBank (accession number KJ957189), the 16s rDNA sequence of *Lactobacillus* JBD301 was used to identify homologous sequences by searching the NCBI GenBank ([www.ncbi.nlm.nih.gov/genbank](http://www.ncbi.nlm.nih.gov/genbank)), the DNA data bank of Japan ([www.ddbj.nig.ac.jp](http://www.ddbj.nig.ac.jp)) and the European Nucleotide Archive ([www.ebi.ac.uk/ ena/](http://www.ebi.ac.uk/%20ena/)). The 16s rDNA sequences of the identified *Lactobacilli* were aligned by ClustalW2 ([www.ebi.ac.uk/Tools/msa/clustalw2](http://www.ebi.ac.uk/Tools/msa/clustalw2)), and a phylogenetic tree was derived using Phylogeny.fr ([www.phylogeny.fr](http://www.phylogeny.fr)) [2].

*2.9. Analysis of FFs A absorption and excretion in mice fed Lactobacilli JBD301*

C57BL/6 female mice (Joongang Experimental Animal Co., Seoul, Korea) were housed at 7 animals per cage with food and water available *ad libitum* under a 12-h light / 12-h dark cycle at 22 ± 2 °C and 55 ± 5% humidity. After 1 week of acclimation, 7-week-old mice were provided a HFD containing 45% kcal as fat (D12451, Research Diets Inc., New Brunswick, NJ, USA) or HFD supplemented with 10^7^ CFU *Lactobacillus* JBD301 for 3 weeks.

For the analysis of FFAs in the intestinal fluid content, mice were killed after 3-week administration of *Lactobacillus* JBD301. Both total fluid contents from small and large intestine were collected immediately and weighed. The gut fluid contents were diluted 5-fold (wt/vol) with sterile saline. Then, the samples in saline were centrifuged at 10,000 × g for 30 min. After centrifugation, the supernatants were collected and analyzed for total FFAs content using the EnzyChrom™ Free Fatty Acid Assay Kit (Bio-Assay Systems).

For the analysis of FFAs absorption and excretion, ^14^C-labeled triolein was fed after 3-week administration of *Lactobacillus* JBD301. The 200 µL of triolein mixture containing [^14^C] triolein (1 µCi/mouse) (PerkinElmer Life Sciences) was administered orally to the *Lactobacillus*-colonized mice. After feeding ^14^C-labeled triolein, blood samples were taken from tail vein at 2, 4, 6, 8, 10, 48, 72 and 96 h, respectively. The fecal samples were also collected daily for 4 days. The blood serum was mixed with equal volume of Solvable™ to measure radioactivities. To measure the radioactivities in the feces, 0.1 g of feces was mixed with 1 mL of Solvable™ (PerkinElmer Life Sciences) at 60 °C with shaking at 900 rpm for 1 h, and the samples were further pulverized by bead homogenizer and the supernatant were collected after centrifugation at 13,000 rpm for 5 min. For decolorization, 20 µL of H_2_O_2_ added to 200 µL of samples with shaking for 10 min at 50 °C, 500 rpm. After centrifugation at 13,000 rpm for 5 min, 100 µL of the supernatants was mixed with 100 µL liquid scintillation cocktail, and the ^14^C activity was determined by Microplate Scintillation Counter (PerkinElmer Life Sciences).

*2.10. Analysis of body weight in DIO mice fed Lactobacilli JBD301*

C57BL/6 female mice (Joongang Experimental Animal Co) were housed at 7 animals per cage with food and water available *ad libitum* under a 12-h light / 12-h dark cycle at 22 ± 2 °C and 55 ± 5% humidity. After 1 week of acclimation, 7-week-old mice were provided a HFD containing 45% kcal as fat (D12451) or HFD supplemented with 10^7^ CFU *Lactobacillus* JBD301 to induce obesity. After 12 weeks on the HFD, the animals were randomly divided into groups that received HFD only (control), HFD supplemented with *Lactobacillus* JBD301 at 10^7^ CFU /day, or HFD supplemented with orlistat (Xenical® at 50 mg/kg diet). Body weight measurements were obtained weekly for 4 weeks.

*2.11. Clinical trial of Lactobacillus JBD301 in overweight/obese human subjects*

A randomized phase 2, double-blind, placebo-controlled human study was conducted with *Lactobacillus* JBD301 in the Clinical Trial Center at the Samsung Medical Center (Seoul, Korea). The research protocol was approved by the Clinical Trial Center at the Samsung Medical Center, and the methods were carried out in accordance with the approved guidelines (SMC No. 2011-04-061-002). This trial was registered with Clinical Research Information Service of Korea as KCT0000452 (<https://cris.nih.go.kr>). The purpose of this study was to evaluate the safety and tolerability of *Lactobacillus* JBD301 and to evaluate the preliminary efficacy of *Lactobacillus* JBD301 compared to placebo for the reduction of body fat or weight in obese adults. The study was designed for Randomized allocation, Placebo Control, Endpoint Classification of Safety/Efficacy, Intervention Model of Parallel Assignment, Masking for Double-Blind (Subject, Investigator) and purpose for treatment of overweight/obesity.
 For the calculation of number of the subjects, the following was assumed; 1) Level of significance, 5% in Two-tailed test, 2) Type 2 error (β) with a 0.2, retaining 80% of the power of the test, 3) The ratio of subject numbers in investigational and control groups as 1, and 4) Comparison of the validity of the evaluation parameters in the investigational and control groups after ingestion of investigational product. To test this, study hypothesis are as follows; H0: μt = μc (Body fat reduction in the test group and that in the control group is the same.), H1: μt ≠ μc (Body fat reduction in the test group and that in the control group is different.). Based on this, the subject number for each group is 44 and 55 subjects should be recruited considering 20% drop-out. Therefore, total number of enrolled subject should be 110.

Study participants were recruited and enrolled for the study if they satisfied the following criteria: 1) written informed consent, 2) men or women 25-65 years of age, and 3) body mass index of 25-35 kg/m^2^. The exclusion criteria included the following: subjects who received antibiotics within 12 weeks prior to the first visit; subjects with current use of medications for body weight reduction (lipase inhibitors, anti-depressants, appetite suppressants), diuretics, contraceptives, steroids, and hormones; subjects who were involved in commercial diet programs or were under a diet formula within 12 weeks prior to the first visit; subjects with uncontrolled hypertension or diabetes; subjects with current medical conditions including cardiac, renal, hepatic, neurovascular, thyroid or parathyroid disorders/diseases; subjects with medical conditions including depression, schizophrenia, psychosis, alcohol abuse or drug abuse; subjects diagnosed with cancer within the past 5 years; subjects with hyper-allergic reactions to components in the testing food; subjects who were planning to reduce body weight by food and exercise during the clinical trial period; and subjects that were pregnant or breastfeeding. The subjects were instructed to maintain their usual diet with no intake of probiotics, antibiotics or agents known to affect body weight. The subjects were withdrawn if they did not complete the scheduled visits or the diet administration.

After enrolment, the subject was accessed by the inclusion/exclusion criteria and then allowed subjects were simply randomized for parallel assignment. Randomization table was generated with simple random number generation using SimpleRNG and was kept in the sealed envelope and delivered to the pharmacist at SMC before the trial. The pharmacist provided to the subject the material which has the same code as the randomization number of the subject. The randomized numbers with allocation concealment was given only to the subject who meets the criteria for inclusion. Allocation concealment was maintained to the clinical investigators including coordinators, care providers, and data collectors as well as to the subjects until the end of this trial for double-blind study.

The study consisted of daily administration of 1 capsule of either *Lactobacillus* JBD301 or placebo for 12 weeks without any dietary restriction. In a capsule of 450 mg, major ingredient, either JBD301 powder or non-dairy coffee creamer, is 12% while other excipients include 40% skim milk, 40% sucrose and 8% ascorbic acid. Among the 110 randomized subjects, experimental subjects within R1001 to R1070 received the low dose of *Lactobacillus* JBD301, 1 × 10^8^ CFU per capsule. Experimental subjects within R1071 to R1110 received the high dose of *Lactobacillus* JBD301, 1 × 10^9^ CFU per capsule. In this study, the subgroup statistical analyses were performed on the subjects with the high dose of *Lactobacillus* JBD301, R1071 to R1110.

The primary outcome from this study was the preliminary efficacy of JBD301 compared to placebo for reduction of abdominal fat or weight in obese adults after administration for 12 weeks. The secondary outcome from this study was the preliminary efficacy of JBD301 compared to placebo for the body fat, body weight, hip circumference, blood lipid profile, fasting blood glucose, blood insulin, and HbA1c in obese adults at 0, 6 and 12 weeks after administration. The adipose tissue area and body fat mass of each subject were measured using computerized tomography (CT) and Dual-Emission X-ray Absorptiometry (DEXA), respectively. For safety measures, abnormal reactions, vital signs, and laboratory tests were measured.

*2.12. Statistical analysis*

The preclinical data were expressed as the mean ± the SEM or the mean ± the SD, as indicated. The statistical comparisons were performed by analysis of variance (ANOVA) tests, unless stated otherwise. All differences were considered statistically significant if p *<* 0.05. Statistical significance is shown as *p < 0.05, **p < 0.01 and ***p < 0.001 versus control. For the clinical data, the statistical analysis was performed using procedures in SAS (Version 9.2) and MedCalc (Version 11.6.0). The models were not adjusted for BMI, age, and sex. Depending on the normality of the underlying data, the Mann-Whitney U test and the Wilcoxon Signed Rank test were used to perform statistical analyses. Unless otherwise stated, a range reported with a mean refers to the standard error of the mean (± SEM). Per protocol analysis was used to determine the sample of participants to be analyzed in this study. The Mann-Whitney U test was used to compare the outcome variables for each assessment time (0 and 12 weeks) and the percentage changes in outcome variables in the two treatment groups (*Lactobacillus* JBD301 vs. placebo). The percentage change in outcome variables at 12 weeks (%) was calculated by the following formula: ((value at 12 weeks – value at 0 week)/value at 0 week) × 100. The Wilcoxon Signed Rank test was used to compare the differences in outcome variables for each assessment time (0, 6 and 12 weeks) and the percentage differences in outcome variables for the two treatment groups (*Lactobacillus* JBD301 vs. placebo). The percentage differences in outcome variables were calculated by the following formula: (value of experimental/value of placebo) × 100. For all comparisons, a significance level of 0.05 (not adjusted for multiple comparisons) was used.

**References**

1. Santhana, R.L. Hing, H.L., Baharudin, O., Teh, H.Z., Aida, S.R., Nor, A.C.P., Vimala, B., Paramsarvaran, S., Sumarani, G. and Hanjeet, K. (2007) Rapid method for transmission electron microscope study of Staphylococcus aureus ATCC 25923. Ann Microscope 7, 102−108.
2. Dereeper, A., Guignon, V., Blanc, G., Audic, S., Buffet, S., Chevenet, F., Dufayard, J.F., Guindon, S., Lefort, V., Lescot, M., Claverie, J.M. and Gascuel, O. (2008) Phylogeny.fr: robust phylogenetic analysis for the non-specialist. Nucleic Acids Res. 36, W465−469.
